# Vibration and Buckling Characteristics of Functionally Graded Graphene Nanoplatelets Reinforced Composite Beams with Open Edge Cracks

**DOI:** 10.3390/ma12091412

**Published:** 2019-04-30

**Authors:** Meifung Tam, Zhicheng Yang, Shaoyu Zhao, Jie Yang

**Affiliations:** 1 School of Engineering, RMIT University, PO Box 71, Bundoora, VIC 3083, Australia; s3407314@student.rmit.edu.au; 2 Guangzhou University-Tamkang University Joint Research Centre for Engineering Structure Disaster Prevention and Control, Guangzhou University, Guangzhou 510006, China; cs.yeung@e.gzhu.edu.cn; 3 School of Civil Engineering, the University of Queensland, St Lucia, Brisbane, QLD 4072, Australia; shaoyu.zhao@uq.edu.au

**Keywords:** graphene nanoplatelets, functionally graded nanocomposites, free vibration, buckling, edge crack

## Abstract

This paper investigates the free vibration and compressive buckling characteristics of functionally graded graphene nanoplatelets reinforced composite (FG-GPLRC) beams containing open edge cracks by using the finite element method. The beam is a multilayer structure where the weight fraction of graphene nanoplatelets (GPLs) remains constant in each layer but varies along the thickness direction. The effective Young’s modulus of each GPLRC layer is determined by the modified Halpin-Tsai micromechanics model while its Poisson’s ratio and mass density are predicted according to the rule of mixture. The effects of GPLs distribution pattern, weight fraction, geometry, crack depth ratio (CDR), slenderness ratio as well as boundary conditions on the fundamental frequency and critical buckling load of the FG-GPLRC beam are studied in detail. It was found that distributing more GPLs on the top and bottom surfaces of the cracked FG-GPLRC beam provides the best reinforcing effect for improved vibrational and buckling performance. The fundamental frequency and critical buckling load are also considerably affected by the geometry and dimension of GPL nanofillers.

## 1. Introduction

Graphene reinforced polymer nanocomposites have been attracting considerable attention from both research and industry communities due to their exceptional mechanical properties [1]. Compared to other reinforcements, such as carbon black (CB) [2], carbon fibers (CFs) [3] and carbon nanotube (CNT) [4], graphene and its derivatives give better performance among these reinforcements. Rafiee et al. [5,6] experimentally found that the Young’s modulus of the epoxy composite increased by 31% when reinforced by graphene nanoplatelets (GPLs) while only a 3% increase was achieved when it was reinforced by CNTs. Tang et al. [7] reported that the graphene reinforced nanocomposite possesses higher strength and fracture toughness when the graphene is highly dispersed in the polymer matrix. Liu et al.’s comparative study [8,9] demonstrated that the mechanical properties of graphene reinforcing alumina ceramic composites is higher than those of monolithic ceramic composites. Lee et al. [10] synthesized functionalized graphene sheet/epoxy nanocomposites for cryotank application and found a significant increase in strength and toughness. Some other notable research works also proved that graphene can significantly increase the elastic stiffness and strength of polymer nanocomposites [11,12,13,14].

In order to make the best use of graphene reinforcements, the concept of functionally graded materials (FGM) [15,16] is introduced into GPLs reinforced composites. Since it is very difficult to fabricate a functionally graded structure with GPLs reinforcement varying continuously and smoothly over thickness direction due to the constraints of current manufacturing technology, Yang and his co-workers [17,18,19] introduced a layer-wise functionally graded nanocomposite beam reinforced by GPLs (FG-GPLRC). Huang et al. [20,21,22] investigated the nonlinear in-plane instability of composite arches reinforced by graphene nanoplatelets and found that the buckling behaviors of FG-GPLRC arches are significantly affected by the GPLRC distribution pattern. Shen et al. [23,24] conducted a series of studies on the nonlinear bending, thermal buckling and post-buckling of functionally graded graphene-reinforced composite laminated beams resting on elastic foundations and discussed the effects of the graphene reinforcement distribution, laminate layer stacking sequence, temperature variation and foundation stiffness on the mechanical behaviors of the beam in detail. Other notable research work on this GPLs reinforced composite can also be found in the literature [25,26,27]. Due to the mechanical advantages of high stiffness and strength, graphene nanocomposites offer huge potential in weight-sensitive applications such as aerospace, automotive and marine structures.

It is well known that a crack in a structure can reduce the stiffness of the structure and may change the mechanical characteristics and also mode shapes of the structure due to the local flexibility introduced by the crack [28,29,30,31,32,33,34]. Lellep et al. [35,36,37] focused on the problem of vibration and optimization of elastic solids with and without a crack. Yang and his co-workers [38,39,40,41] investigated the free and nonlinear vibration, buckling and post-buckling of a cracked FGM beam and discussed the effect of cracks on the mechanical behavior of a FGM beam in detail. However, to the best of the authors’ knowledge, no previous work has been done on cracked graphene reinforced nanocomposite structures, including any of its mechanical characteristics. Hence, this paper aims to numerically study the free vibration and buckling behaviors of FG-GPLRC beams with edge cracks by using the finite element method. The Halpin-Tsai micromechanics model and rule of mixture are employed to determine the effective Young’s modulus, Poisson’s ratio and mass density, respectively. A comprehensive parametric study is conducted to examine the effects of the GPL distribution pattern, weight fraction, geometry, crack depth ratio (CDR), slenderness ratio and boundary conditions of the beam on the characteristics of the fundamental frequency and the critical buckling load.

## 2. Effective Material Properties of GPLRC

**Figure 1:**
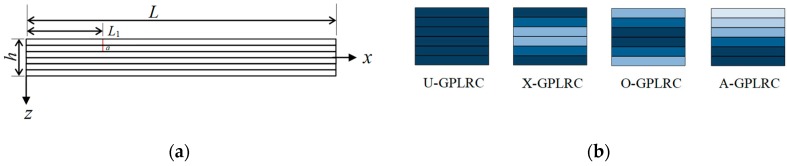
(**a**) Configuration and coordinate system of a graphene nanoplatelets reinforced composite (GPLRC) cracked beam; (**b**) GPL distribution patterns.

A FG-GPLRC beam with the dimensions of length *L*, thickness *h* and width *b* consists of multilayer isotropic polymer materials reinforced by GPLs that uniformly disperses and randomly orients in each layer, containing an edge crack of depth *a* located at a distance *L*_1_ from the left end as shown in Figure 1a. Each GPLRC layer has equal thickness and perfectly bonds with each other. As shown in Figure 1b, the darker color represents a higher content of GPL nanofillers allocated in this layer. For the U-GPLRC beam, all layers are uniformly dispersed with the same content of GPL nanofillers. The X-GPLRC beam has higher content of GPL nanofillers on the top and bottom layers of GPL nanofillers. The O-GPLRC beam has the opposite distribution of GPL nanofillers toward the X-GPLRC beam. The GPL nanofillers in the A-GPLRC beam gradually increase from the top layer to the bottom layer.

Among the micromechanical models used to predict the effective elastic modulus of GPLRC such as the Halpin-Tsai model [42], Mori-Tanaka model [43,44] and Voigt model [45], etc., the Halpin-Tsai model is used in the present work since it has been experimentally demonstrated that this model gives a better prediction for the elastic modulus of GPLRC than other models [46]. Hence, the effective Young’s modulus of each GPLRC layer is evaluated using the Halpin-Tsai model as follows,

(1) $$E=\frac{3E_{m}}{8}\frac{1+\xi_{L}\eta_{L}V_{\mathrm{GPL}}^{\left(k\right)}}{1-\eta_{L}V_{\mathrm{GPL}}^{\left(k\right)}}+\frac{5E_{m}}{8}\frac{1+\xi_{T}\eta_{T}V_{\mathrm{GPL}}^{\left(k\right)}}{1-\eta_{T}V_{\mathrm{GPL}}^{\left(k\right)}}$$

where the parameters $\eta_{L}$ and $\eta_{T}$ are expressed as follows,

(2) $$\eta_{L}=\frac{(E_{\mathrm{GPL}}/E_{m})-1}{(E_{\mathrm{GPL}}/E_{m})+\xi_{L}},\;\eta_{T}=\frac{(E_{\mathrm{GPL}}/E_{m})-1}{(E_{\mathrm{GPL}}/E_{m})+\xi_{T}}$$

in which *E*_GPL_ and *E*_m_ are moduli of the GPL and matrix, respectively. The geometry factors *ξ*_L_ and *ξ*_T_ of GPLs considered in Equation (1) are defined by

(3) $$\xi_{L}=2(a_{\mathrm{GPL}}/t_{\mathrm{GPL}}),\;\xi_{L}=2(b_{\mathrm{GPL}}/t_{\mathrm{GPL}})$$

where *a*_GPL_, *b*_GPL_ and *t*_GPL_ are the length, width and thickness of GPLs, respectively, and therefore

(4) $$\xi =2(a_{\mathrm{GPL}}/b_{\mathrm{GPL}})(b_{\mathrm{GPL}}/t_{\mathrm{GPL}})$$

where *a*_GPL_/*b*_GPL_ is the aspect ratio and *b*_GPL_/*t*_GPL_ is the width-to-thickness ratio. The *k*-th layer volume fraction of GPLs $V_{\mathrm{GPL}}^{\left(k\right)}$ considered in Equation (1) takes the form of

(5) $$\text{U-GPLRC}:\;V_{\mathrm{GPL}}^{\left(k\right)}=V_{\mathrm{GPL}}^{*},$$

(6) $$\text{X-GPLRC}:\;V_{\mathrm{GPL}}^{\left(k\right)}=2V_{\mathrm{GPL}}^{*}|2k-N_{L}-1|/N_{L},$$

(7) $$\text{O-GPLRC}:\;V_{\mathrm{GPL}}^{\left(k\right)}=2V_{\mathrm{GPL}}^{*}\left(1-|2k-N_{L}-1|/N_{L}\right),$$

(8) $$\text{A-GPLRC}:\;V_{\mathrm{GPL}}^{\left(k\right)}=V_{\mathrm{GPL}}^{*}\left(2k-1\right)/N_{L},$$

where the total number of layers for the beam is *N*_L_ and *k* = 1, 2, …, *N*_L_. The total GPL volume fraction $V_{\mathrm{GPL}}^{*}$ is expressed by

(9) $$V_{\mathrm{GPL}}^{*}=\frac{W_{\mathrm{GPL}}}{W_{\mathrm{GPL}}+\left(\rho_{\mathrm{GPL}}/\rho_{m}\right)\left(1-W_{\mathrm{GPL}}\right)},$$

where *W*_GPL_ is the total GPL weight fraction in the whole beam.

Since the Halpin-Tsai model can only give the elastic modulus, the mass density ρ and Poisson’s ratio υ of GPLs reinforced composite materials are determined by employing the rule of mixture in this paper [23,24,25].

(10) $$\rho =\rho_{m}V_{m}+\rho_{\mathrm{GPL}}V_{\mathrm{GPL}}$$

(11) $$\upsilon =\upsilon_{m}V_{m}+\upsilon_{\mathrm{GPL}}V_{\mathrm{GPL}}$$

where *ρ*_GPL_ and *ρ*_m_ are mass densities of the GPL and the matrix, *υ*_GPL_ and *υ*_m_ are Poisson’s ratios of the GPL and the matrix, respectively. The volume fractions *V*_GPL_ and *V*_m_ are related by *V*_m_ + *V*_GPL_ = 1.

## 3. Finite Element Implementation

**Figure 2:**
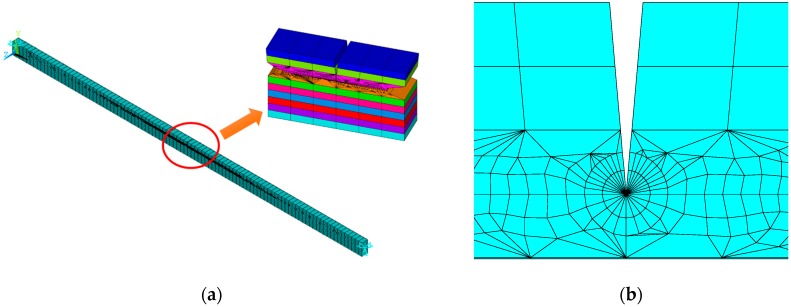
ANSYS models for the cracked functionally graded-graphene nanoplatelets reinforced composite (FG-GPLRC) beam. (**a**) Mesh of beam, (**b**) mesh of crack tip.

In this paper, ANSYS 14.5 (ANSYS, Inc., Cannonsburg, PA, USA) is employed as the finite element (FE) package to compute the natural frequencies and the buckling loads of FG-GPLRC beams with edge cracks. The crack is assumed to locate perpendicularly to the top surface along the width direction of the GPLRC layer and has a V-shaped. In finite element simulation, a structural solid element PLANE183 is used to model the cracked FG-GPLRC beam model, and the multi-layered GPLRC modelled by PLANE183 element is perfectly glued layer-by-layer. The mesh grid of the beam and around the crack tip is shown in Figure 2. For the GPLRC layers with a crack, singular elements as shown in Figure 2b are used to mesh the area in the vicinity of the crack to model the stress singularity at the crack tip. For the GPLRC layers without crack, 200 rectangular elements with the same thickness as a single GPLRC layer are adopted to mesh the perfect GPLRC layer.

For the finite element method, the model of the cracked FG-GPLRC beam is discretized into an assemblage of discrete finite elements, which are interconnected at the nodal points on element boundaries. The displacement field can be represented using the nodal displacements, which is approximated over each finite element. The equations of motion can then be formulated using the displacement-based formulation in conjunction with the principle of virtual displacements. These equations of motion can be written as [47],

(12) $$M\ddot{U}+\left(K_{e}+K_{\sigma }\right)U=0,$$

where overdots indicate differentiation with respect to time, M, $K_{e}$ and $K_{\sigma }$ are the mass matrix, bending stiffness matrix and geometric stiffness matrix that are dependent on the effective Young’s modulus, Poisson’s ratio and mass density determined from the Halpin-Tsai model (Equations (1)–(9)) and rule of mixture (Equations (10) and (11)) and contain crack parameters of the FG-GPLRC beam, respectively. It should be noted that the geometric stiffness matrix depends on the pre-stressed state in the structure as well.

For the harmonic free vibration, Equation (12) yields

(13) $$\left(K_{e}-\omega^{2}M\right)U=0,$$

where ω represents the fundamental frequency of the FG-GPLRC beam.

For the elastic buckling, the critical buckling load can be obtained from Equation (12) by neglecting the inertia terms. An eigenvalue equation can be derived as

(14) $$\left(K_{e}+\lambda K_{\sigma }\right)U=0,$$

from which the critical buckling load of the beam can be obtained by solving the equation det(**K** + *λ***K_σ_**) = 0, where the scale parameter *λ* represents the critical buckling load.

It should be noted that due to the multi-layered nature of the beam, the homogeneous element formulation which assumes constant material properties within each element is used in the present study. Such an approximation requires uniform and fine meshing along the beam thickness. Otherwise, a graded element formulation [48] in which the material properties are graded at element level is more appropriate and should be used for more accurate analysis.

## 4. Results and Discussion

### 4.1. Verification

The size and mechanical parameters of the isotropic homogeneous Timoshenko beam employed in this study are: length *L* = 0.2 m, height *h* = 0.0078 m, Young’s modulus *E* = 216 GPa, density *ρ* = 7850 kg/m^3^ and Poisson ratio *v* = 0.28. The first natural frequencies of a clamped-free (C-F) beam with an open edge crack of depth *a*/*h* = 0.2 at different locations (*L*_1_/*L* = 0.2, 0.4, 0.6) are compared with that of an intact beam in Table 1. The results obtained by the present method show good agreement with the analytical results obtained by Ke et al. [38].

Table 2 compares the critical buckling load and dimensionless fundamental frequency for an intact C-C FG-X-GPLRC beam based on the present method with the corresponding results for an intact FG-GPLRC based on the first shear deformation theory [49]. Again, the results also achieve excellent agreement.

In what follows, the free vibration behaviors and elastic buckling characteristics of edge cracked FG-GPLRC beams with three types of boundary conditions, namely, clamped-free (C-F); hinged-hinged (H-H), and clamped-clamped (C-C) are investigated. The cracked beam has a cross-section with *b* × *h* = 0.03 m × 0.06 m and has an edge crack with depth *a*. Each GPLRC layer of the beam is made from a mixture of epoxy and GPLs with *a*_GPL_ = 2.5 µm, *b*_GPL_ = 1.5 µm and *t*_GPL_ = 1.5 nm. The Young’s moduli and mass densities of the epoxy and GPLs are 3 GPa, 1200 kg/m^3^ and 1010 GPa, 1062.5 kg/m^3^, and the Poisson’s ratios *υ* of the epoxy and GPLs are 0.186 and 0.34, respectively. Moreover, it was concluded in our previous work [17,18,19] that an ideal functionally graded beam which is continuous in material properties and composition can be accurately modelled using a multilayer FG-GPLRC beam with *N*_L_ = 10. Thus, *N*_L_ = 10 is used in all of the following numerical analyses.

### 4.2. Free Vibration Analysis

**Figure 3:**
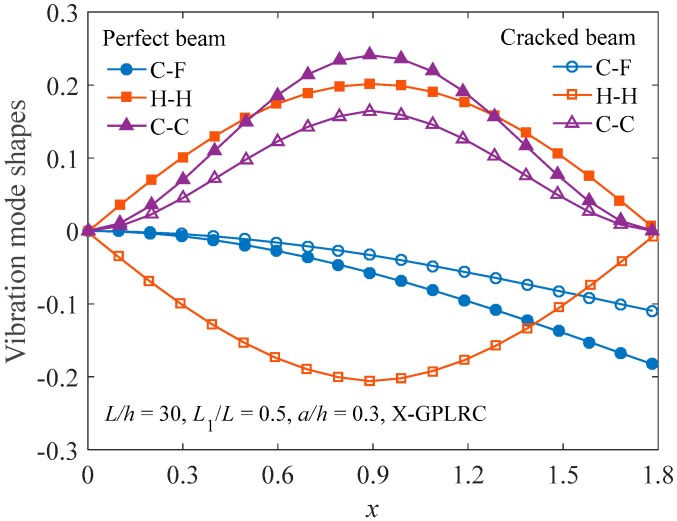
Fundamental vibration mode shapes of intact and cracked FG-X-GPLRC beams.

Figure 3 illustrates the fundamental vibration mode shapes of functionally graded X-GPLRC beams in the presence of a crack. The results for the perfect beams without a crack are also provided for comparison. Based on the fact that the mode shape can be arbitrarily scaled, it is evident that virtually the mode shape is not affected by the edge crack.

Table 3 gives information about the dimensionless fundamental frequencies $\omega_{1}/\omega_{10}$ for C-C cracked FG-GPLRC beams with CDR (*a*/*h*) = 0.3. Noted that $\omega_{1}$ is the fundamental frequency of cracked FG-GPLRC beams, and $\omega_{10}$ is the fundamental frequency of pure epoxy beam without a crack. As shown in Table 3, the fundamental frequencies for C-C cracked FG-GPLRC beams with different weight fractions and distribution patterns of GPLs are tabulated. A higher bending stiffness of the cracked FG-X-GPLRC beam can be obtained by dispersing more GPLs filler in two sides of the layers. As a result, the beam with this kind of GPLs distribution patterns (FG-X) has a greater fundamental frequency followed by the FG-U-, FG-A- and FG-O-GPLRC beams. In addition, there is an increase in all of the fundamental frequencies with the increase in the GPLs weight fraction. The fundamental frequencies of cracked FG-GPLRC beams for different crack positions are much greater than those of the pure epoxy beams, illustrating the considerable reinforcement effect of GPLs.

**Figure 4:**
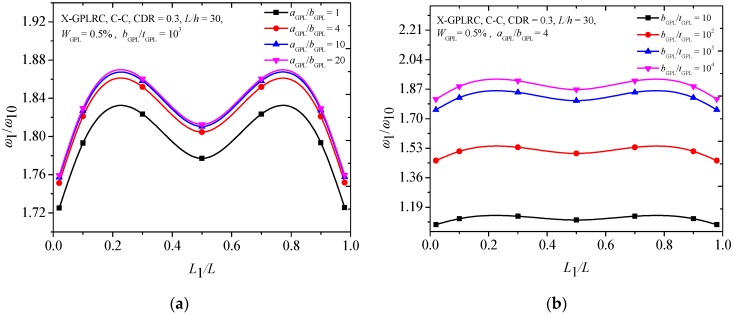
Effects of (**a**) aspect ratio (**b**) width-to-thickness ratio on the fundamental frequency of the C-C cracked FG-X-GPLRC beam.

**Figure 5:**
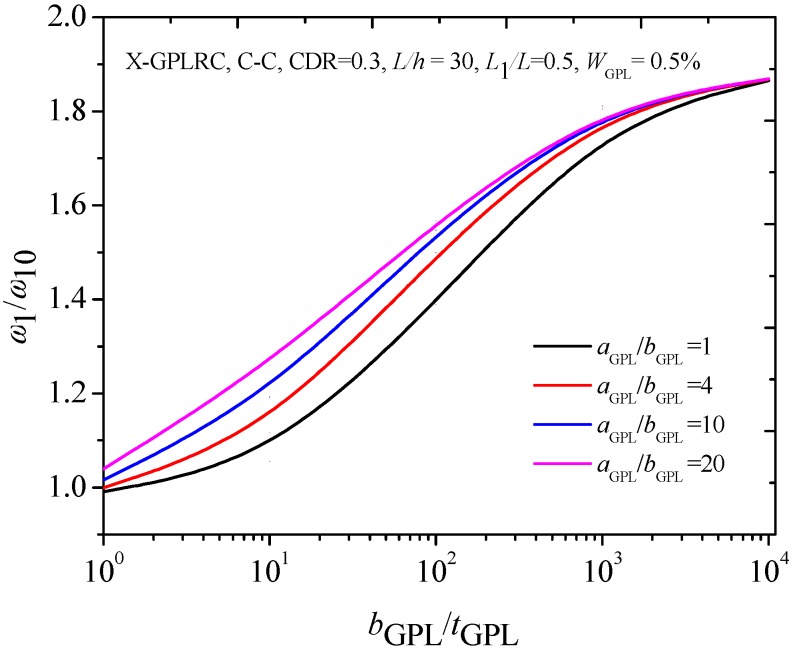
Effect of GPL geometry on fundamental frequency of C-C cracked FG-X-GPLRC beam.

Figure 4 gives information about the effect of the geometry and dimension of GPLs on the fundamental frequencies of the C-C cracked FG-X-GPLRC beam by using the parameters as the aspect ratio (*a*_GPL_/*b*_GPL_) and width-to-thickness ratio (*b*_GPL_/*t*_GPL_). When the width *b*_GPL_ of GPL is kept constant, a larger value of aspect ratio (*a*_GPL_/*b*_GPL_) shows that a single graphene layer with larger surface area, and a greater magnitude width-to-thickness ratio (*b*_GPL_/*t*_GPL_) reflects fewer single graphene layers. The numerical results show that the fundamental frequencies increase with the *a*_GPL_/*b*_GPL_ and *b*_GPL_/*t*_GPL_, and the increases are limited after *a*_GPL_/*b*_GPL_ = 4 and *b*_GPL_/*t*_GPL_ = 10^3^, respectively. It was also found that smaller fundamental frequencies appear when the crack is located near two ends and at the mid-span of the beam. Figure 5 shows that the fundamental frequency ratios increase as both *a*_GPL_/*b*_GPL_ and *b*_GPL_/*t*_GPL_ increase. While the changes are much less pronounced when *b*_GPL_/*t*_GPL_ is close to 10^3^, beyond this the fundamental frequency remains almost unchanged.

Table 4 lists the dimensionless fundamental frequencies of the C-C cracked FG-X-GPLRC beam with different CDRs where CDR = 0.0 indicates a beam without crack. It is found that the existence of the crack is remarkable for the change of fundamental frequencies of the beam, and the fundamental frequencies decrease as the CDR increases. The reason is that there is a more drastic decline in stiffness due to the rise of crack depth ratios in the beam.

**Figure 6:**
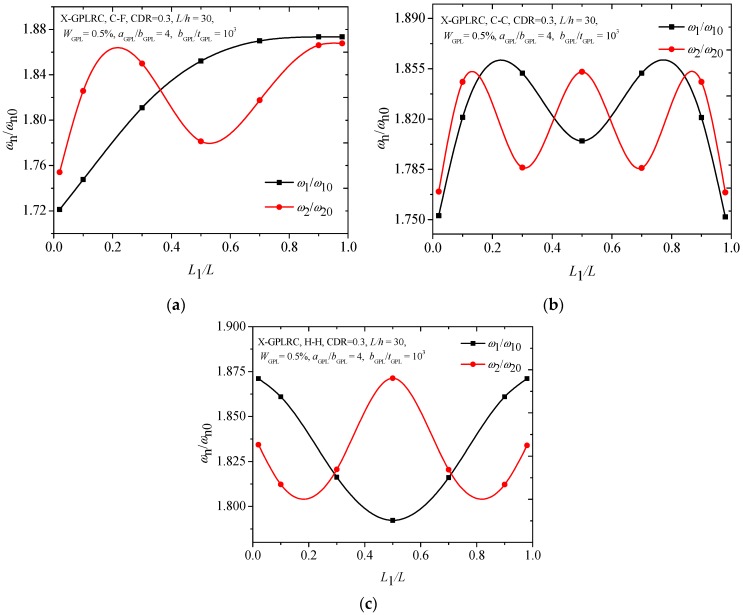
First two frequencies ratios of cracked FG-X-GPLRC beams with different boundary conditions: (**a**) C-F, (**b**) C-C, (**c**) H-H.

**Figure 7:**
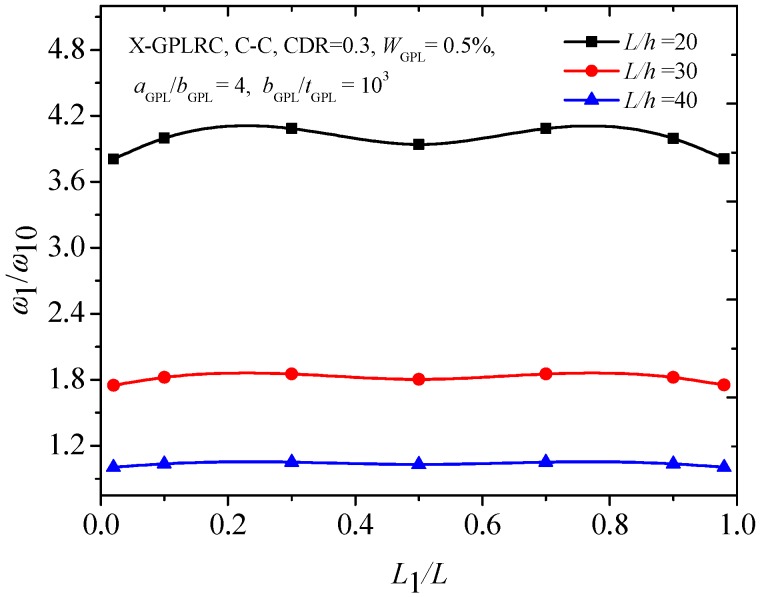
Effect of slenderness ratio on fundamental frequency of C-C cracked FG-X-GPLRC beam.

Figure 6 compares the first two frequencies of the FG-X-GPLRC with different boundary conditions. It can be found that variations in the second frequencies of the cracked FG-X-GPLRC beam with different boundary conditions seem to be much more complicated than those for the fundamental frequencies. From the Figure 6a,b, the lowest frequencies for the first two frequencies of the C-C and C-F beams are found to be at the same locations. However, for the H-H beam, it has the lowest ratio at *L*_1_/*L* = 0.5 for the fundamental frequency, and at *L*_1_/*L* = 0.2, 0.8 for the second frequency. Figure 7 shows that the C-C cracked FG-X-GPLRC beam with a lower slenderness ratio has a much greater fundamental frequency due to its much greater bending rigidity.

### 4.3. Buckling Analysis

**Figure 8:**
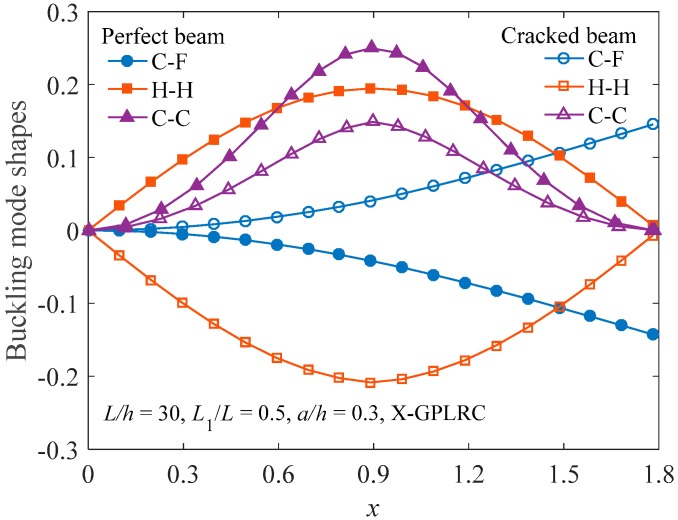
Comparison of buckling mode shapes of intact and cracked FG-X-GPLRC beams.

**Figure 9:**
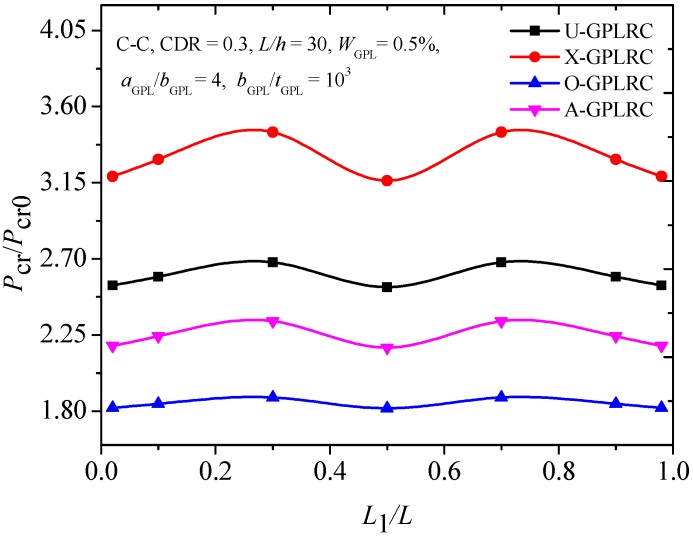
Effects of GPL distribution patterns on critical buckling load of cracked FG-GPLRC beams.

Figure 8 plots the buckling mode shape of the FG-X-GPLRC beam with or without an open edge crack. Similar to the vibration mode shapes in Figure 3, the buckling mode shape is not sensitive to the edge crack. Figure 9 shows the critical buckling load for cracked C-C FG-GPLRC beams with different distribution patterns of GPL. The FG-X-GPLRC beam gives the highest critical buckling load because layers at two sides of the beam, where the greater normal bending stress appears, distribute more GPLs. Hence, the FG-X-GPLRC beam produces the best reinforcing effect among the four kinds of beams. Table 5 shows that the critical buckling loads of the FG-X-GPLRC beam are clearly higher than those of the pure epoxy beam (*W*_GPL_ = 0.0%) and increase as GPL weight fraction increases.

**Figure 10:**
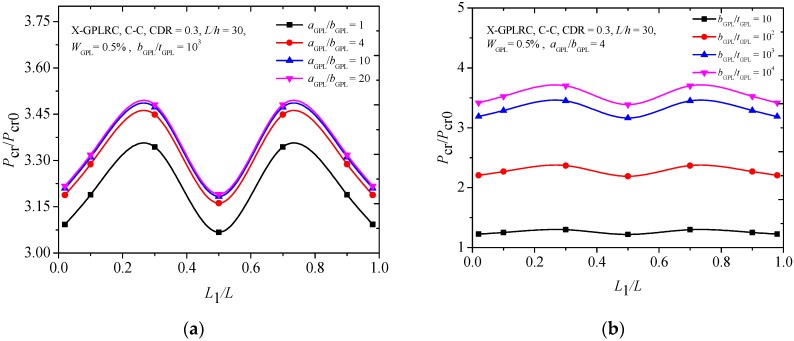
Effects of (**a**) aspect ratio and (**b**) width-to-thickness ratio on the critical buckling load of cracked FG-X-GPLRC beam.

Figure 10 compares the effects of GPLs geometry and dimensions on the critical buckling load of the FG-X-GPLRC beam with an edge crack. It is observed that the changes of the critical buckling load are more sensitive to the GPLs width-to-thickness ratio (*b*_GPL_/*t*_GPL_) (Figure 10b) than those of the aspect ratio (*a*_GPL_/*b*_GPL_) (Figure 10a), although the critical buckling loads increase as both *b*_GPL_/*t*_GPL_ and *a*_GPL_/*b*_GPL_. It is also seen that the smaller critical buckling loads for the C-C FG-X-GPLRC beam occur when the edge crack is located at the mid-span or near the end of the beam, which is similar to the results of the free vibration analysis (Figure 4).

**Figure 11:**
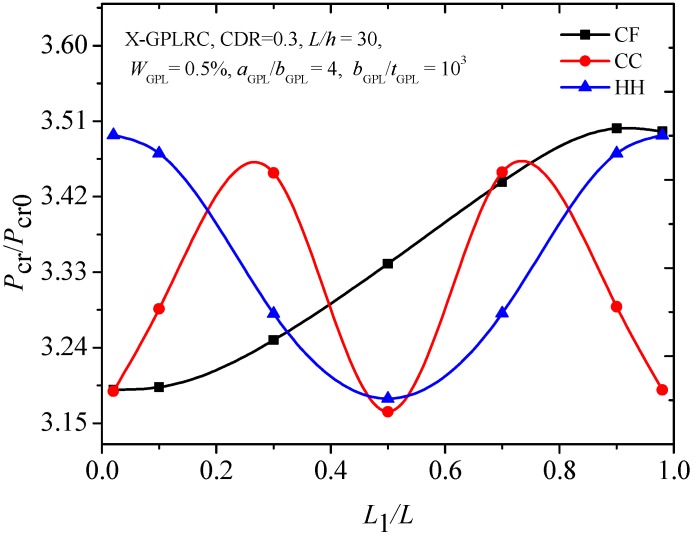
Critical buckling load of cracked FG-X-GPLRC beams with different boundary conditions.

Figure 11 illustrates the critical buckling loads of C-C, C-F and H-H beams with the edge crack at different crack locations. It is clear that there are symmetric critical buckling loads with respect to the crack location curves of C-C and H-H beams, which have geometrical symmetry. For the C-F beam, the highest and lowest critical buckling loads occur when the edge crack is located at the free end and restrained end, respectively.

Table 6 lists the critical buckling loads of C-C cracked FG-X-GPLRC beam with various CDR *a*/*h* and slenderness ratio *L*/*h*. It can be found that the CDR has a significant influence on the critical buckling loads of the cracked FG-X-GPLRC beam, and the critical buckling loads decrease as CDR increases. It is noted that a lower slenderness ratio leads to a much greater critical buckling load as expected.

## 5. Conclusions

This paper illustrates the free vibration and buckling behaviors of graphene nanoplatelets reinforced functionally graded beams with edge cracks by using finite element modelling. The effective material properties of GPLRC were determined by the Halpin-Tsai micromechanics model. Numerical results are shown in both graphical and tabular forms to examine the influences of GPLs distribution pattern, weight fraction, GPLs geometry, crack depth and location, the slenderness ratio as well as the boundary conditions of the beam on the fundamental frequency and critical buckling load of the cracked FG-GPLRC beam. It was found that the fundamental frequency and critical buckling load drop with the increase in crack depth, and the lowest fundamental frequency and critical buckling load of C-C and H-H beams are found when the crack is located in the midspan. However, for the C-F beam, the lowest values are determined when the crack is located at the end. The cracked FG-GPLRC beam with more GPLs distributed near the surface, which has a greater natural frequency and critical buckling load than the other distribution patterns, makes more effective use of GPL reinforcements. It was also found that the influences of the geometry and size of GPL nanofillers are quite significant but limited when the *b*_GPL_/*t*_GPL_ of GPLs is higher than 10^3^. This paper also discussed and analyzed the influences of the slenderness ratio and boundary conditions through illustrative numerical examples.

## Figures and Tables

**Table 1:** Comparison of fundamental frequency for a cracked isotropic homogenous clamped-free (C-F) beam.

Comparison Results	*L*_1_/*L* = 0.2	*L*_1_/*L* = 0.4	*L*_1_/*L* = 0.6	Intact Beam
Present	1019.9	1029.9	1035.2	1036.9
Ke et al. [38]	1020.98	1029.853	1034.932	1037.0106

**Table 2:** Comparison of buckling and free vibration results for an intact clamped-clamped (C-C) FG-X-GPLRC beam.

Comparison Results	*P*_cr_	*w*_1_
Present	0.0090	0.3359
Wu et al. [49]	0.0089	0.3350

(*N* = 11, *N*_L_ = 10, *L*/*h* = 30, *W*_GPL_ = 0.3%).

**Table 3:** Dimensionless fundamental frequencies $\omega_{1}/\omega_{10}$ of cracked C-C FG-GPLRC beams. (*L*/*h* = 30, *a*_GPL_/*b*_GPL_ = 4, *b*_GPL_/*t*_GPL_ = 10^3^, CDR = 0.3).

Distribution Patterns	*W*_GPL_	*L*_1_/*L*		
		0.2	0.5	0.8
U-GPLRC	0.1%	1.1567	1.1328	1.1567
	0.3%	1.4200	1.3906	1.4200
	0.5%	1.6417	1.6074	1.6417
X-GPLRC	0.1%	1.2229	1.1929	1.2229
	0.3%	1.5762	1.5310	1.5762
	0.5%	1.8628	1.8047	1.8627
O-GPLRC	0.1%	1.0856	1.0666	1.0856
	0.3%	1.2393	1.2212	1.2393
	0.5%	1.3758	1.3574	1.3758
A-GPLRC	0.1%	1.1447	1.1189	1.1447
	0.3%	1.3604	1.3277	1.3604
	0.5%	1.5332	1.4946	1.5332
Pure epoxy	0.0%	0.9994	0.9785	0.9994

**Table 4:** Dimensionless fundamental frequencies $\omega_{1}/\omega_{10}$ of cracked FG-X-GPLRC beams with different CDR (*L*/*h* = 30, *a*_GPL_/*b*_GPL_ = 4, *b*_GPL_/*t*_GPL_ = 10^3^, *W*_GPL_= 0.5%, C-C).

CDR	*L*_1_/*L*				
	0.02	0.2	0.5	0.8	0.98
0.0	1.8643	1.8643	1.8643	1.8643	1.8643
0.1	1.8513	1.8642	1.8584	1.8642	1.8513
0.2	1.8127	1.8637	1.8392	1.8637	1.8127
0.3	1.7513	1.8628	1.8047	1.8627	1.7518

**Table 5:** Critical buckling loads of cracked C-C FG-X-GPLRC beams with different weight fraction (*L*/*h* = 30, *a*_GPL_/*b*_GPL_ = 4, *b*_GPL_/*t*_GPL_ = 10^3^, CDR = 0.3).

*W*_GPL_	*L*_1_/*L*				
	0.02	0.2	0.5	0.8	0.98
0.0%	0.9425	0.9941	0.9394	0.9941	0.9429
0.1%	1.3980	1.4885	1.3917	1.4885	1.3988
0.3%	2.2980	2.4727	2.2820	2.4728	2.2980
0.5%	3.1880	3.4529	3.1618	3.4530	3.1880

**Table 6:** Critical buckling loads of C-C cracked FG-X-GPLRC beams (*L*_1_/*L* = 0.5, *a*_GPL_/*b*_GPL_ = 4, *b*_GPL_/*t*_GPL_ = 10^3^, *W*_GPL_ = 0.5%).

CDR	*L*/*h*		
	20	30	40
0.0	7.6778	3.4855	1.9753
0.1	7.5725	3.4537	1.9615
0.2	7.2426	3.3531	1.9172
0.3	6.6581	3.1618	1.8336
